# Comparison of venetoclax and azacitidine versus a cytarabine‐based treatment for patients with acute myeloid leukaemia in first relapse after chemotherapy. A real‐world study of the DATAML‐IPC‐AURAML consortium

**DOI:** 10.1111/bjh.70815

**Published:** 2026-09-04

**Authors:** Pierre‐Yves Dumas, Sarah Bertoli, Emilie Bérard, Clémence Santana, Maël Heiblig, Thibaut Leguay, Suzanne Tavitian, Estelle Borgne, Anne‐Charlotte de Grande, Audrey Sarry, Ziyad Acheaibi, Laetitia Largeaud, Morgane Sighieri, Emilie Klein, Emmanuelle Tavernier, Marie‐Anne Hospital, Martin Carré, Gaspar Aspas Requena, Anne Banos, Norbert Vey, Arnaud Pigneux, Christian Récher, Sylvain Garciaz

**Affiliations:** ^1^ Univ. Bordeaux, INSERM, BRIC, U1312, CHU Bordeaux, Service d'Hématologie Clinique et de Thérapie Cellulaire, CHU Bordeaux Bordeaux France; ^2^ Université de Toulouse, CHU de Toulouse, Institut Universitaire du Cancer de Toulouse Oncopole, Service d'Hématologie Toulouse France; ^3^ Department of Clinical Epidemiology and Public Health CERPOP, INSERM‐University of Toulouse III, Toulouse University Hospital (CHU) Toulouse France; ^4^ Department of Hematology CH de Valence Valence France; ^5^ Department of Hematology CHU Lyon Sud Lyon France; ^6^ Aix‐Marseille University, INSERM U1068, CNRS, Institut Paoli‐Calmettes, CRCM Marseille France; ^7^ Laboratoire d'Hématologie Biologique, CHU Bordeaux Bordeaux France; ^8^ Department of Hematology CHU de St Etienne Saint‐Priest‐en‐Jarez France; ^9^ Department of Hematology CHU de Grenoble Grenoble France; ^10^ Department of Hematology CHU de Montpellier Montpellier France; ^11^ Department of Hematology CHG de Bayonne Bayonne France

**Keywords:** acute myeloid leukaemia, azacitidine, cytarabine, first relapse, real‐life, venetoclax

## Abstract

Complete remission (CR) rates for patients with acute myeloid leukaemia (AML) treated with intensive chemotherapy (IC) have increasingly improved. Yet, relapse remains a concern with a dismal prognosis. In this report, results are presented of a retrospective analysis comparing salvage therapy based on intermediate (I) or high (H)‐dose cytarabine (DAC) (*n* = 203) and venetoclax‐azacitidine (VEN‐AZA) (*n* = 114) for patients with AML in first relapse. Patients (median age 60 years, interquartile range [IQR] 48–67) had reached CR1 after a standard 7 + 3 (*n* = 305) or CPX351 schedule (*n* = 12). I/HDAC‐based and VEN‐AZA salvage, respectively, resulted in CR2 in 66.0% and 66.7% of the patients (*p* = 0.96). Allogeneic haematopoietic stem cell transplantation (allo‐HSCT) was performed for 115 patients in CR2 and 18 in failure. Relapse‐free survival was 13.2 (IQR 7.0‐not reached [NR]) and 13.7 (IQR 7.8‐NR) months while overall survival (OS) was 14.5 (IQR 6.2‐NR) and 12.3 months (IQR 4.8‐NR; *p* = 0.41) for patients who received I/HDAC‐based salvage and VEN‐AZA respectively. In multivariable analysis, relapsing patients ≥60 years with low/intermediate‐risk cytogenetics and VEN‐AZA had a better OS (*p* = 0.002), while I/HDAC was more efficient for those with poor risk cytogenetics and < 60‐year‐old (*p* = 0.002). VEN‐AZA salvage therapy thus appears promising compared with conventional I/HDAC‐based IC; this question warrants evaluation in randomized studies.

## INTRODUCTION

Front‐line treatment for patients with acute myeloid leukaemia (AML) still largely relies on intensive chemotherapy (IC), combining cytarabine and an anthracycline, although some molecular anomalies can benefit from targeted therapy. If complete remission with complete (CR) or incomplete (CRi) haematological recovery can now be reached in more than 70% of patients, discrepancies remain, based on age, comorbidities and individual molecular landscapes. Patients eligible for allogeneic haematopoietic stem cell transplantation (allo‐HSCT) have a reduced risk of relapse and prolonged survival if they get transplanted. However, many patients still relapse, mostly as they could not benefit from allo‐HSCT. Their prognosis, except in small categories such as AML with Core‐Binding Factor alteration, is dismal and salvage therapy depends on each patient ability to receive IC. Whenever possible, intermediate (I) or high (H)‐dose cytarabine (DAC)‐based regimens are commonly used,^1^ leading to 40%–50% of CR2.^2^

Since the VIALE‐A trial^3^ and subsequent authorization of the B‐cell lymphoma 2 (BCL‐2) inhibitor venetoclax (VEN) in 2020, this molecule has been combined with the hypomethylating agent azacitidine (AZA) as front‐line therapy for patients (mostly elderly) ineligible for IC.^4^ In addition, the VEN‐AZA combination is commonly used off‐label to treat molecular^5^ or morphological^6^, ^7^, ^8^, ^9^, ^10^, ^11^ relapse, as well as primary induction failure, in fit patients.^12^ In the study reported here, AML patients in first relapse after front‐line IC, who received either I/HDAC‐based intensive salvage or VEN‐AZA, were compared.

## PATIENTS AND METHODS

Patients with AML, aged 18–75 years at diagnosis, were eligible for this retrospective study if they had achieved CR/CRi after a single induction course for newly diagnosed AML, and subsequently experienced a first morphological relapse according to the European LeukemiaNet (ELN) 2022 definition. They also should have received either an I/HDAC‐based IC regimen or VEN‐AZA as salvage therapy. I/HDAC‐based IC included intermediate dose cytarabine (IDAC) at 1–1.5 g/m^2^/injection, high‐dose (HDAC) cytarabine at 3 g/m^2^/injection, I/HDAC with gemtuzumab ozogamycin, I/HDAC with anthracycline or FLAG‐idarubicin regimens. VEN was administered orally once daily at 400 mg after a 3‐day ramp up, or at 100 mg in case of concomitant azoles administration. Patients received AZA at 75 mg/m^2^ subcutaneously from day (d)1 until d7 of each cycle or from d1 to d5 and d8 to d9. During the first cycle, VEN was given until d14–21 according to each centre usual practices. After remission, VEN was given for 7–21 days according to previous adverse events.

Exclusion criteria were addition of a third drug to the VEN‐AZA salvage schedule (so‐called ‘triplet’) or salvage treatment for molecular relapse. Patients with primary induction failure were not included. Written informed consent was obtained from all patients in accordance with the Declaration of Helsinki, allowing for the collection of clinical data in the anonymized French Toulouse‐Bordeaux DATAML (Commission Nationale de l'Informatique et des Libertés #DR‐2015‐529), Institut Paoli‐Calmettes (IRB2003‐010) and AURAML (IRB00013204) registries, for this retrospective collection of real‐world data.

Considering statistical analyses, continuous variables are expressed as medians, interquartile range (IQR) and range. Categorical variables are expressed as numbers and percentages of non‐missing data. Comparisons between groups used likewise parametric (chi‐squared or Student) or non‐parametric (Fisher exact or Mann Whitney) tests when necessary. Relapse‐free survival (RFS) and overall survival (OS) were, respectively, calculated from the date of first relapse treatment to the date of second relapse, death or last follow‐up and to death or last follow‐up. Kaplan–Meier survival curves were drawn. Cumulative incidence of relapse (CIR) was calculated from the date of first relapse treatment to the date of second relapse or last follow‐up. Death without relapse was considered as a competing event of relapse. Differences in survival were evaluated by the log‐rank (for RFS and OS) or Gray test (for CIR). In multivariable analysis, differences in CR2/CRi2 rate and OS between I/HDAC versus VEN‐AZA groups were tested using, respectively, a logistic regression or a Cox model. Potential confounding factors were sex, AML status, cytogenetic prognosis, *FLT3*‐ITD mutation, *NPM1* mutation, I/HDAC consolidation, first‐line (1 L) allo‐HSCT, age at relapse, time to relapse, ECOG (Performance status) at relapse, white blood cell (WBC) at relapse and 2 L allo‐HSCT (for OS exclusively) as a time‐dependent variable. When the linearity hypothesis was not respected, continuous variables were transformed into ordered data using previously published thresholds or the quartiles method. Variables were considered to be introduced in multivariable analyses, together with the I/HDAC versus VEN‐AZA groups, if they yielded a *p*‐value <0.20 in univariable analysis. A stepwise analysis was then applied. Interactions between independent covariates and I/HDAC versus VEN‐AZA groups were tested in the final models. As recommended, in case of a significant interaction (*p* < 0.10), analyses were stratified. *p*‐values lower than 0.05 were considered for statistical significance. Statistical analyses were performed on STATA® version 18.0 (STATA Corp., College Station, TX).

## RESULTS

Between 1 January 2015 and 30 September 2023, 317 patients with AML from 9 French centres were identified as having been treated for a first relapse after IC, respectively, 203 (64%) with I/HDAC and 114 (36%) with VEN‐AZA. Disease and first‐line treatment characteristics are reported in Table 1. Globally, 176/317 (55.5%) patients were male and the median age at diagnosis of the cohort was 60 years (IQR 48–67). Overall, 276 (87.1%) and 41 (12.9%) patients presented with de novo or secondary AML, respectively. At diagnosis, 10.6% had an *IDH1* mutation and 14.9% an *IDH2* mutation without difference between the two groups. Conversely, *NPM1* mutation was present in 41.8% versus 15.5% of patients and *TP53* mutation in 0.0% versus 15.3% of patients, in the I/HDAC‐based salvage group versus the VEN‐AZA salvage group respectively. Induction chemotherapy was a classical 7 + 3 combination for 305 (96.2%) patients and the liposomal CPX‐351 formulation for 12 (3.8%). Post‐remission strategy was I/HDAC‐based for 227 (75.4%) patients, while 3 (1.0%) received CPX‐351. Finally, 76 (24.0%) patients underwent allo‐HSCT in first CR.

**TABLE 1:** Characteristics at diagnosis and front‐line treatment.

	Total	I/HDAC‐based	VEN‐AZA	*p*‐value
	*n* = 317 (100%)	*n* = 203 (64.0%)	*n* = 114 (36.0%)	
Age (years) at diagnosis				<0.001
Median (IQR)	60.0 (48.0–67.0)	57.0 (45.0–64.0)	63.5 (56.0–70.0)	
Range	19.0–75.0	19.0–73.0	20.0–75.0	
Gender: *n* (%)				0.687
Male	176 (55.5)	111 (54.7)	65 (57.0)	
Female	141 (44.5)	92 (45.3)	49 (43.0)	
AML status: *n* (%)				<0.001
De novo	276 (87.1)	188 (92.6)	88 (77.2)	
Secondary AML^a^	41 (12.9)	15 (7.4)	26 (22.8)	
Cytogenetic risk: *n* (%)^b^				<0.001
Favourable	32 (10.2)	28 (13.8)	4 (3.6)	
Intermediate	235 (74.8)	158 (77.8)	77 (69.4)	
Adverse	47 (15.0)	17 (8.4)	30 (27.0)	
*FLT3*‐ITD: *n* (%)				0.127
No	259 (88.1)	158 (85.9)	101 (91.8)	
Yes	35 (11.9)	26 (14.1)	9 (8.2)	
*NPM1* mutation: *n* (%)				<0.001
No	200 (68.0)	107 (58.2)	93 (84.5)	
Yes	94 (32.0)	77 (41.8)	17 (15.5)	
*IDH1* mutation: *n* (%)				0.140
No	194 (89.4)	95 (86.4)	99 (92.5)	
Yes	23 (10.6)	15 (13.6)	8 (7.5)	
*IDH2* mutation: *n* (%)^c^				0.425
No	211 (85.1)	106 (84.8)	105 (85.4)	
Yes	37 (14.9)	19 (15.2)	18 (14.6)	
*TP53* mutation: *n* (%)				<0.001
No	137 (91.3)	65 (100.0)	72 (84.7)	
Yes	13 (8.7)	0 (0.0)	13 (15.3)	
Front‐line IC: *n* (%)				<0.001
7 + 3	305 (96.2)	202 (99.5)	103 (90.4)	
CPX‐351	12 (3.8)	1 (0.5)	11 (9.6)	
Post‐remission: *n* (%)				<0.001
I/HDAC	227 (75.4)	162 (83.1)	65 (61.3)	
Other	74 (24.6)	33 (16.9)	41 (38.7)	
Allo‐HSCT in CR1: *n* (%)				<0.001
Yes	76 (24.0)	33 (16.3)	43 (37.7)	
Time between allo‐HSCT in CR1 and relapse (months)				0.413
Median (IQR)	12.5 (4.2–31.4)	17.7 (5.5–33.5)	11.5 (3.9–31.4)	
Range	0.79–166.2	0.99–67.9	0.79–166.2	
Time to relapse (months)				0.754
Median (IQR)	11.2 (6.3–21.4)	11.1 (6.5–20.1)	11.5 (6.1–22.4)	
Range	0.03–225.4	0.26–160.5	0.03–225.4	
Time to relapse: *n* (%)				0.680
≤6 months	71 (22.4)	44 (21.7)	27 (23.7)	
>6 months	246 (77.6)	59 (78.3)	87 (76.3)	

Abbreviations: AML, acute myeloid leukaemia; CR, complete response; HSCT, haematopoietic stem cell transplantation; I/HDAC, intermediate/high‐dose cytarabine; IC intensive chemotherapy; IQR, interquartile range; ITD, internal tandem duplication; VEN‐AZA, venetoclax‐azacitidine.

^a^ Non‐de novo AML (post‐myelodysplastic syndrome (MDS) *n* = 35, post‐myeloproliferative neoplasm (MPN) *n* = 7, post‐chronic myelomonocytic leukemia (CMML) *n* = 48, therapy‐related *n* = 10, others *n* = 10).

^b^ According to MRC 2010 classification.

^c^ 31 *IDH2* R140 and 6 *IDH2* R172.

Based on the salvage therapy, baseline differences between the I/HDAC and VEN‐AZA groups were the older age at diagnosis of patients in the latter, who also had more secondary AML, were more often given CPX‐351 at induction, and received less I/HDAC‐based consolidation programme. Cytogenetic and molecular landscapes also differed significantly between the two groups, with less patients at poor cytogenetic risk in the I/HDAC salvage group. Finally, significantly more patients of the VEN‐AZA salvage group received allo‐HSCT (*p* < 0.001) in CR1 (Table 1). First relapse occurred after a median delay of 11.2 months (IQR 6.3–21.4) post‐CR1/CRi1, but within 6 months for 71 (22.4%) patients, without difference between the two groups (*p* = 0.75).

Patient characteristics and treatment at first relapse are reported in Table 2. Patients in the VEN‐AZA group were older (*p* < 0.0001) and had a lower white blood cell count (WBC) at relapse (*p* = 0.018). Early death ≤ d30 in the whole population occurred for 12 (3.8%) patients without any difference related to salvage treatment (*p* = 0.5). Comparable CR2/CRi2 rates were obtained with the use of I/HDAC (134/203, 66.0%) or VEN‐AZA (70/114; 66.7%; *p* = 0.91). Patients of the VEN‐AZA group who reached CR2/CRi2 received a median number of 4 (IQR 2–9; range 1–21) cycles.

**TABLE 2:** Patient characteristics at relapse and response to salvage treatment.

	Total	I/HDAC‐based	VEN‐AZA	*p*‐value
	*n* = 317 (100%)	*n* = 203 (64.0%)	*n* = 114 (36.0%)	
Age (years) at relapse				0.001
Median (IQR)	61.0 (49.0–68.0)	59.0 (46.0–66.0)	65.0 (57.0–72.0)	
Range	20.0–83.0	20.0–76.0	21.0–83.0	
ECOG: *n* (%)				0.090
0–1	234 (86.3)	142 (89.3)	92 (82.1)	
≥2	37 (13.7)	17 (10.7)	20 (17.9)	
WBC (10^9^/L)				0.018
Median (IQR)	3.1 (1.9–7.1)	3.5 (2.1–7.8)	2.7 (1.8–6.1)	
Range	0.3–265.7	0.6–265.7	0.3–70.0	
Response: *n* (%)				0.908
CR2/CRi2	204 (66.2)	134 (66.0)	70 (66.7)	
Failure	104 (33.8)	69 (34.0)	35 (33.3)	
Allo‐HSCT *n* (%)				<0.001
No	183 (57.7)	98 (48.3)	85 (74.6)	
Yes^b^	134 (42.3)	105 (51.7)	29 (25.4)	
Early death: *n* (%)^a^				
<Day 30	12 (3.8)	9 (4.4)	3 (2.6)	0.547
<Day 60	32 (10.1)	18 (8.9)	14 (12.3)	0.332

Abbreviations: Allo‐HSCT, allogeneic haematopoietic stem cell transplantation; CR, complete response; ECOG, performance status; I/HDAC, intermediate/high‐dose cytarabine; IQR, interquartile range; VEN‐AZA, venetoclax‐azacitidine; WBC, white blood cells count.

^a^ From date of relapse treatment.

^b^ 115 patients in CR2/CRi2, 1 morphological free state and 18 in treatment failure.

In multivariable analysis, male sex (adjusted odds ratio [aOR] 2.26; 95% confidence interval [CI] 1.31–3.90; *p* = 0.003) and a longer time to relapse (>6 months; aOR 7.11, 95% CI 3.76–13.46; *p* < 0.0001) remained statistically significant. Moreover, significant interactions were observed between salvage treatment and cytogenetic risk (*p* = 0.002), on the one hand, and between salvage treatment and age at relapse (*p* = 0.085), on the other hand. This led to stratify multivariable analysis on cytogenetics results and age at relapse. In patients with a favourable/intermediate karyotype and age at relapse ≥60 years, VEN‐AZA treatment yielded a significantly higher CR2/CRi2 rate than I/HDAC (aOR 2.66, 95% CI 1.17–6.05, *p* = 0.019). Conversely, in patients with adverse cytogenetic risk and age at relapse ≥60 years, VEN‐AZA was significantly and independently associated with a lower CR2/CRi2 rate than I/HDAC (aOR 0.22, 95% CI 0.05–0.98; *p* = 0.046). Similarly, in patients with adverse cytogenetic risk and age at relapse <60 years, VEN‐AZA was significantly and independently associated with a lower CR2/CRi2 rate than I/HDAC (aOR 0.08, 95% CI 0.01–0.41; *p* = 0.003). In patients with favourable/intermediate karyotype and age at relapse <60 years, the CR2/CRi2 rate was not significantly different between salvage treatments (aOR 0.91, 95% CI 0.33–2.49; *p* = 0.855).

Overall, 134 patients received allo‐HSCT after relapse, significantly more being treated with I/HDAC than with VEN‐AZA (*p* < 0.0001), whether in CR2/CRi2 (*n* = 90 [67.2%] vs. 25 [35.7%]) or in failure (*n* = 15 [21.7%] vs. 3 [8.6%]). Finally, 96 patients developed a second relapse, without any difference according to the first relapse salvage treatment (47.0% I/HDAC vs. 47.1%, VEN‐AZA).

There was no difference in CIR between the two salvage schedules, with, respectively, 37.1% and 41.2% of relapsed patients at 1 year for I/HDAC and VEN‐AZA (*p* = 0.53) treatments (Figure S1). Interactions between salvage treatment and cytogenetic risk (*p* = 0.792) or age at relapse (*p* = 0.976) were not significant regarding CIR. No difference in RFS after CR2/CRi2 was observed between the I/HDAC and VEN‐AZA treatment groups (Figure S2) with respective medians of 13.2 (IQR 7.0‐not reached [NR]) and 13.7 (IQR 7.8‐NR) months and 1‐year RFS of 51.2% (95% CI 42.2–59.5) and 56.5% (95% CI 43.3–67.7; *p* = 0.92). Interactions between salvage treatment and cytogenetic risk (*p* = 0.615) or age at relapse (*p* = 0.515) were not statistically significant for RFS.

With a median follow‐up from relapse to treatment initiation of 36 months (IQR 17.0–36.0), median OS, respectively, 14.5 months (IQR 6.2‐NR) for patients who received I/HDAC‐based salvage and 12.3 months (IQR 4.8‐NR; *p* = 0.41) for those treated with VEN‐AZA (Figure 1). There was no difference either in 1‐year OS at, respectively, 53.0% (95% CI 45.8–59.7) and 52.4% (95% CI 42.2–61.7).

**FIGURE 1:**
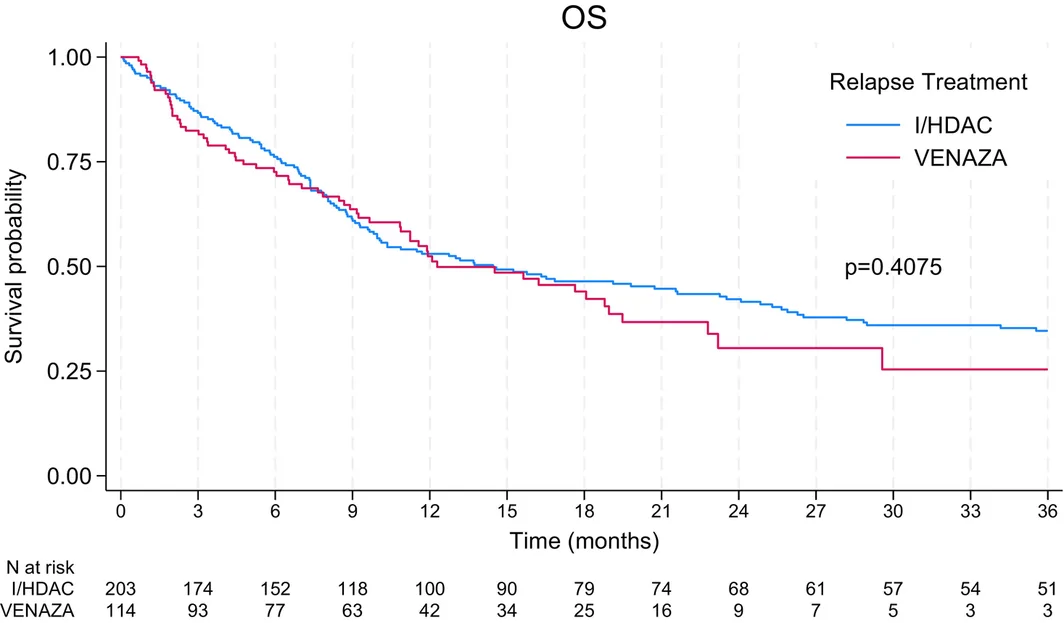
OS from relapse treatment initiation.

Multivariable analysis (Tables S1 and S2) retained de novo AML, time to relapse >6 months, WBC at relapse <30 × 10^9^/L and 2 L allo‐HSCT as predictive of longer OS. Moreover, significant interactions were observed between the type of salvage treatment and both cytogenetic risk (*p* = 0.003) and age at relapse (*p* = 0.010). This led to stratify multivariable analysis on these variables (Table S2). In patients with a favourable/intermediate karyotype and aged ≥60 years at relapse, VEN‐AZA treatment yielded a significantly better OS than I/HDAC (adjusted hazard ratio [aHR] 0.47, 95% CI 0.30–0.76; *p* = 0.002) (Figure 2A) while there was no difference for younger patients (aHR 1.13, 95% CI 0.64–1.99; *p* = 0.685) (Figure 2B). Conversely, in patients with adverse cytogenetic risk, VEN‐AZA was significantly and independently associated with shorter OS in patients <60 years after relapse (aHR 4.11, 95% CI 1.69–10.0; *p* = 0.002) (Figure 2C), while there was no difference for older patients (aHR 1.73, 95% CI 0.78–3.86; *p* = 0.178) (Figure 2D). Of note, patients who received I/HDAC‐based salvage therapy were also significantly more transplanted than those who received VEN‐AZA, whether they had high‐risk (52.9% vs. 10.0%, *p* = 0.004) or favourable/intermediate (51.6% vs. 29.6%, *p* = 0.001) cytogenetics. Among transplanted patients, 105 had received I/HDAC (4 with ECOG ≥2), while all 29 transplanted patients treated with VEN‐AZA had an ECOG <2. Among non‐transplanted patients, 98 were treated with I/HDAC, including 13 with an ECOG ≥2. Conversely, 23.8% of non‐transplanted patients treated with VEN‐AZA had an ECOG ≥2. Sixteen patients received 2 allografts, 10 in the I/HDAC‐based and 6 in the VEN‐AZA‐based salvage groups. Excluding these patients from OS prognostic factor analysis did not modify results.

**FIGURE 2:**
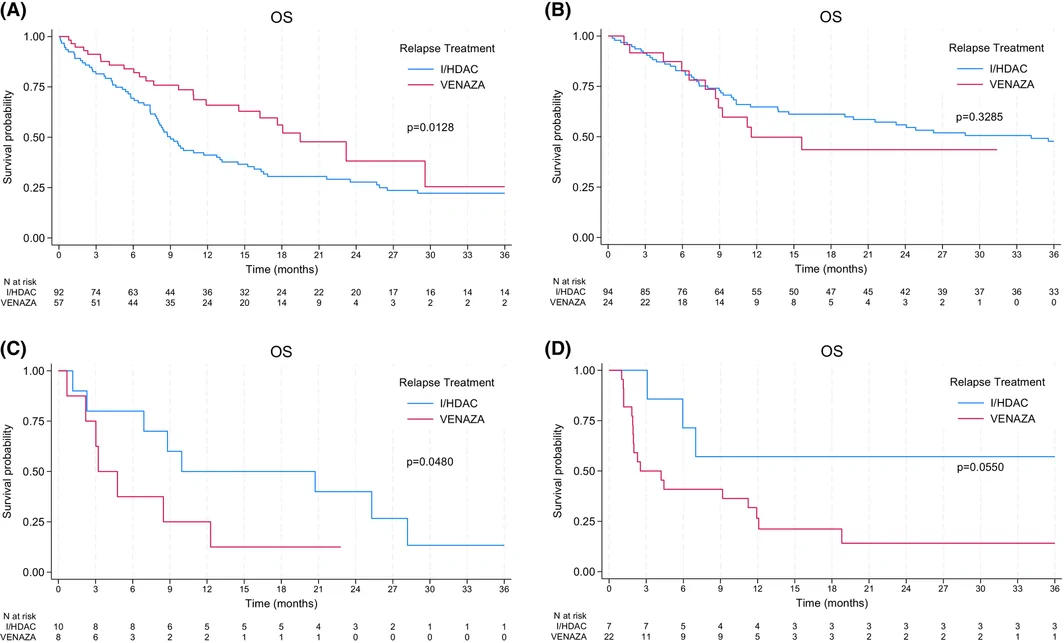
(A) OS in patients with favourable or intermediate cytogenetic risk and age at relapse ≥60 years; (B) OS in patients with favourable or intermediate cytogenetic risk and age at relapse <60 years; (C) OS in patients with adverse cytogenetic risk and age at relapse <60 years; (D) OS in patients with adverse cytogenetic risk and age at relapse ≥60 years.

## DISCUSSION

This original study reports on a comparison of different salvage therapies for patients with AML in first relapse. Indeed, current recommendations are more directly suggesting allo‐HSCT^13^ and do not really compare pre‐transplant strategies. This is especially true in real life, as including patients in clinical trials remains the most recommended attitude.^13^

Here, the retrospective analysis of real‐world registry data demonstrates that the less intensive combination of VEN‐AZA yielded similar response rates and outcomes as the I/HDAC IC schedule. Investigator choices appear to have been driven by patient age (reversed proportions of patients over 60 in the two groups, with more of them treated with VEN‐AZA) and thus, likely, comorbidities even if this could not be explored. Patients treated with I/HDAC, besides being younger and probably fitter, moreover had de novo AML, favourable cytogenetics and *NPM1* mutations, all associated with better outcome.^14^, ^15^, ^16^ Nevertheless, these factors did not impact survivals, suggesting that applying less intensive schedules to probably less fit patients was a good choice.

One confounding factor could be the fact that more patients in the VEN‐AZA group had received allo‐HSCT during first‐line therapy. However, they still did relapse and could have ended up with a far worse outcome. As a consequence, more patients in the I/HDAC group received allo‐HSCT after relapse, yet without impact on a better or worse outcome compared to patients receiving VEN‐AZA.

Broadly, median OS at 12–14 months, whatever the group of salvage treatment, compares favourably with a previous randomized study in relapse or refractory situation.^17^ This is however possibly driven by the exclusion of primary refractory patients with AML.

The most important conclusion of this non‐randomized study is the very interesting OS rate, particularly in the group of older patients over 60, also linked to the exclusion of refractory patients. These results suggest, with the limitation of the sample size, that cytogenetics should be taken into account. VEN‐AZA appeared to confer a meaningful OS advantage in patients ≥60 years with favourable or intermediate cytogenetic risk. Conversely, patients <60 years with adverse‐risk cytogenetics benefited from I/HDAC as second‐line therapy. Although our results do not suggest any meaningful worsening, the width of the CIs highlights the need for cautious interpretation and for larger studies to confirm these observations, notably regarding the imbalance between the two treatment groups, particularly with regard to *TP53* mutations.

In conclusion, this real‐world retrospective study provides results from a currently lacking comparison of I/HDAC versus VEN‐AZA therapy as second‐line treatment for patients in first relapse. It indicates that both strategies are effective and highlights the fact that cytogenetic risk and age could help for decision‐making between both strategies. Moreover, it emphasizes the positive impact of allo‐HSCT, even after relapse.

## AUTHOR CONTRIBUTIONS

**Pierre‐Yves Dumas:** Conceptualization; investigation; writing – original draft; methodology; validation; writing – review and editing; formal analysis; project administration; supervision; data curation. **Estelle Borgne:** Investigation. **Sarah Bertoli:** Investigation. **Clémence Santana:** Data curation; investigation. **Anne‐Charlotte de Grande:** Data curation. **Emilie Bérard:** Conceptualization; methodology; formal analysis; writing – review and editing. **Marie‐Anne Hospital:** Investigation. **Maël Heiblig:** Investigation. **Morgane Sighieri:** Investigation. **Audrey Sarry:** Data curation. **Anne Banos:** Investigation. **Emilie Klein:** Investigation. **Laetitia Largeaud:** Investigation. **Sylvain Garciaz:** Investigation; writing – review and editing; formal analysis. **Christian Récher:** Investigation; writing – review and editing; formal analysis. **Arnaud Pigneux:** Investigation. **Thibaut Leguay:** Investigation. **Ziyad Acheaibi:** Data curation. **Suzanne Tavitian:** Investigation. **Martin Carré:** Investigation. **Gaspar Aspas Requena:** Investigation. **Norbert Vey:** Investigation. **Emmanuelle Tavernier:** Investigation.

## FUNDING INFORMATION

No specific funding.

## CONFLICT OF INTEREST STATEMENT

P.‐Y.D. reports honoraria or research support to institution from Novartis, Servier, Bristol Myers Squibb, Astellas, Daiichi‐Sankyo, AbbVie, Jazz Pharmaceuticals, Janssen, AstraZeneca, Roche. S.B. reports honoraria, support for attending meetings or research support to institution from AbbVie, Astellas, Servier, Bristol Myers Squibb, Jazz Pharmaceuticals, J&J, Novartis. M.H. reports honoraria or research support to institution from Servier, Bristol Myers Squibb, Astellas, Daiichi‐Sankyo, AbbVie. C.R. reports a consulting or advisory role with AbbVie, Daiichi‐Sankyo, Janssen, Servier, received research funding from AbbVie, Iqvia and Servier and support for attending meetings and/or travel from AbbVie, Daiichi‐Sankyo, Novartis and Servier. S.G. declares a consulting or advisory role with AbbVie, Astellas, BMS‐Celgene, Jazz Pharmaceuticals, Imcheck and Servier and received travel grants from Gilead and Sanofi. G.A.R reports honoraria or research support to institution from Servier, Bristol Myers Squibb and AbbVie. E.B., C.S., T.L., S.T., E.B., A.‐C.D.G., A.S., Z.A., L.L., M.S., E.K., E.T., M.‐A.H., M.C., A.B., N.V. and A.P. declare no disclosures.

## ETHICS STATEMENT

This study used information recorded in a consortium database as patients were treated in individual centres. All patients provided informed consent for data collection.

## Supporting information


**Figure S1.** Cumulative incidence of relapse.
**Figure S2**. Relapse‐free survival.
**Table S1**. Univariable analyses of factors affecting OS.
**Table S2**. Multivariable analyses of factors affecting OS.

## Data Availability

Some deidentified data are available upon reasonable request from the corresponding author, Pierre‐Yves Dumas (pierre-yves.dumas@u-bordeaux.fr); data sharing will require a detailed proposal and a data transfer agreement must be signed.
