# Supplementary material for: Comparison of venetoclax and azacitidine versus a cytarabine‐based treatment for patients with acute myeloid leukaemia in first relapse after chemotherapy. A real‐world study of the DATAML‐IPC‐AURAML consortium
Source: Br J Haematol. 2026 Sep 4;209(5):2160–7. doi: 10.1111/bjh.70815 (PMC13748379; doi:10.1111/bjh.70815)
Supplement: Supplementary file 1 [file BJH-209-2160-s001.docx]

**Comparison of venetoclax and azacitidine *versus* a cytarabine-based treatment for patients with acute myeloid leukemia in first relapse after chemotherapy. A real-world study of the** **DATAML-IPC-AURAML consortium.**

**Supplementary material**

Supplementary Figure S1. Cumulative incidence of relapse

Supplementary Figure S2. Relapse-free survival

**Supplementary Table S1. Univariable analyses of factors affecting OS.**

| **Variable** | **Events/Population (%)** | | **HR (95%CI)** | | **p value** |
| --- | --- | --- | --- | --- | --- |
| **Univariable analyses** |  |  | |  |  |
| Group |  | |  | |  |
| I/HDAC | 124/203 (61) | | 1 | |  |
| VEN-AZA | 63/114 (55) | | 1.14 (0.84-1.55) | | 0.408 |
| Sex |  | |  | |  |
| Male | 95/176 (54) | | 1 | |  |
| Female | 92/151 (61) | | 1.22 (0.92-1.63) | | 0.167 |
| AML status |  | |  | |  |
| de novo | 155/276 (56) | | 1 | |  |
| secondary | 32/141 (23) | | 2.02 (1.38-2.97) | | **<0.0001** |
| Cytogenetic risk |  | |  | |  |
| favourable | 10/32 (59) | | 1 | | **<0.0001**^e^ |
| intermediate | 139/235 (59) | | 2.40 (1.26-4.56) | |  |
| adverse | 37/47 (79) | | 4.04 (2.01-8.14) | |  |
| *FLT3*-ITD mutation |  | |  | |  |
| No | 152/259 (61) | | 1 | |  |
| Yes | 23/35 (71) | | 1.11 (0.71-1.72) | | 0.647 |
| *NPM1* mutation |  | |  | |  |
| No | 121/200 (60) | | 1 | |  |
| Yes | 54/94 (57) | | 0.82 (0.60-1.13) | | 0.230 |
| 1L Allo-HSCT |  | |  | |  |
| No | 135/241 (55) | | 1 | |  |
| Yes | 52/76 (68) | | 1.43 (1.04-1.07) | | **0.029** |
| Time to relapse ^a^ |  | |  | |  |
| ≤ 6 months | 55/71 (77) | | 1 | |  |
| > 6 months | 132/246 (54) | | 0.41 (0.30-0.56) | | **<0.0001** |
| Time to relapse ^b^ |  | |  | |  |
| ≤ 6 months | 55/71 (77) | | 1 | | **<0.0001**^e^ |
| > 6 - ≤ 11 months | 50/83 (60) | | 0.53 (0.36-0.77) | |  |
| > 11 - ≤ 21 months | 41/81 (51) | | 0.36 (0.24-0.54) | |  |
| > 21 months | 41/82 (50) | | 0.36 (0.24-0.54) | |  |
| Age at relapse |  | |  | |  |
| < 60 years | 73/138 (53) | | 1 | |  |
| ≥ 60 years | 114/179 (64) | | 1.53 (1.13-2.05) | | **0.005** |
| ECOG at relapse |  | |  | |  |
| 0-1 | 131/234 (56) | | 1 | |  |
| 2-3 | 26/37 (70) | | 2.06 (1.35-3.14) | | **0.001** |
| WBC at relapse |  | |  | |  |
| < 30x10^9^/L | 142/242 (59) | | 1 | |  |
| ≥ 30 x10^9^/L | 14/20 (70) | | 1.68 (0.97-2.91) | | 0.066 |
| Previous I/HDAC ^c^ |  | |  | |  |
| No | 50/74 (67) | | 1 | |  |
| Yes | 126/227 (55) | | 0.73 (0.53-1.02) | | 0.067 |
| Allo-HCT after salvage ^d^ |  | |  | |  |
| No | 52/98 (53) | | 1 | |  |
| Yes | 39/115 (34) | | 0.38 (0.27-0.54) | | **<0.0001** |

^a^ Akaike's information criterion (AIC)=1932.8; ^b^ AIC=1932.3; ^c^ as consolidation program in front line; ^d^ time-dependent variable; ^e^ overall p-value for the variable treated as a categorical variable; I/HDAC, intermediate/high dose cytarabine; VEN-AZA, venetoclax–azacitidine; HR: Hazard Ratio; CI: Confidence Interval

**Supplementary Table S2. Multivariable analyses of factors affecting OS.**

| **Variable** | | **HR (95%CI)** | **p value** | |
| --- | --- | --- | --- | --- |
| **Interaction between salvage treatment and cytogenetic risk** | | | 0.003 | |
| **Interaction between salvage treatment and age at relapse** | | | 0.010 | |
| **Patients with non-adverse cytogenetic risk** **and age at relapse < 60 years** | | | |  |
| VEN-AZA |  | 1.13 (0.64-1.99) | 0.685 | |
| Secondary AML |  | 1.94 (1.27-2.97) | 0.002 | |
| Time to relapse >6 months | | 0.35 (0.25-0.49) | <0.001 | |
| WBC at relapse ≥30x10^9^/L | | 2.49 (1.37-4.54) | 0.003 | |
| Allo-HCT after salvage* | | 0.41 (0.28-0.60) | <0.001 | |
| **Patients with non-adverse cytogenetic risk and age at relapse ≥ 60 years** | | | | |
| VEN-AZA |  | 0.47 (0.30-0.76) | 0.002 | |
| Secondary AML |  | 1.94 (1.27-2.97) | 0.002 | |
| Time to relapse >6 months | | 0.35 (0.25-0.49) | <0.001 | |
| WBC at relapse ≥30x10^9^/L | | 2.49 (1.37-4.54) | 0.003 | |
| Allo-HCT after salvage* | | 0.41 (0.28-0.60) | <0.001 | |
| **Patients with adverse cytogenetic risk and age at relapse < 60 years** | | | |  |
| VEN-AZA |  | 4.11 (1.69-10.0) | 0.002 | |
| Secondary AML |  | 1.94 (1.27-2.97) | 0.002 | |
| Time to relapse >6 months | | 0.35 (0.25-0.49) | <0.001 | |
| WBC at relapse ≥30x10^9^/L | | 2.49 (1.37-4.54) | 0.003 | |
| Allo-HCT after salvage* | | 0.41 (0.28-0.60) | <0.001 | |
| **Patients with adverse cytogenetic risk and age at relapse ≥ 60 years** | | | | |
| VEN-AZA |  | 1.73 (0.78-3.86) | 0.178 | |
| Secondary AML |  | 1.94 (1.27-2.97) | 0.002 | |
| Time to relapse >6 months | | 0.35 (0.25-0.49) | <0.001 | |
| WBC at relapse ≥30x10^9^/L | | 2.49 (1.37-4.54) | 0.003 | |
| Allo-HCT after salvage* | | 0.41 (0.28-0.60) | <0.001 | |

* time-dependent variable; VEN-AZA, venetoclax–azacytidine; HR: Hazard Ratio; CI: Confidence Interval
